# Calling the Steps in Development's Genetic Square Dance

**DOI:** 10.1371/journal.pbio.0020204

**Published:** 2004-07-13

A single, fertilized egg divides into apparently identical daughter cells. As these twins divide again and again, differences emerge among their progeny, establishing segments that will distinguish back from front and head from tail in the growing embryo. Development of segments—and, later, distinct tissues—requires a carefully coordinated square dance of gene expression machinery. Proteins coded by special genes called transcription factors call out the steps by binding to DNA to block or encourage expression of specific genes. With to-the-minute timing, transcription factors call other genes into action to produce the proteins that will determine cell fate. Developing organisms express different transcription factors at specific times and locations to coordinate the changes that make some cells head, others tail; one, a neuron, another, muscle.[Fig pbio-0020204-g001]

**Figure pbio-0020204-g001:**
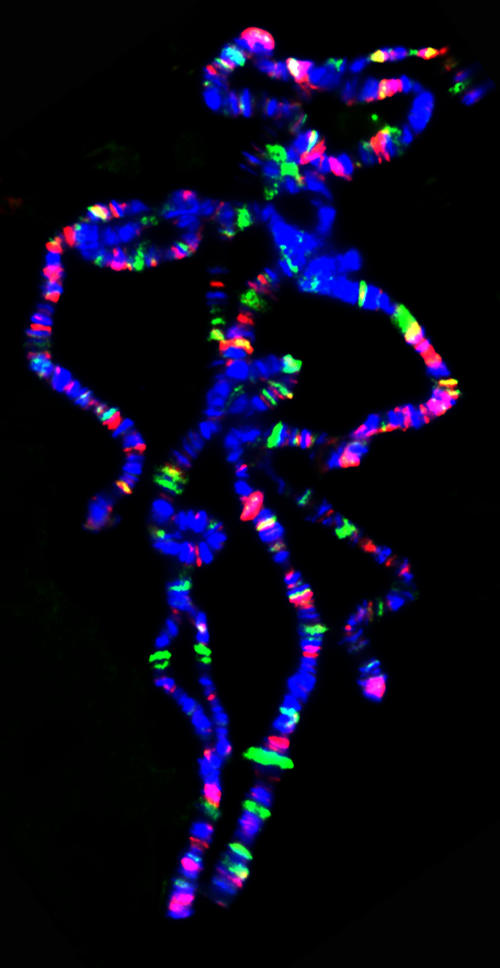
Polytene chromosomes (blue) stained for Hairy (green) and Groucho (red) binding

A transcription factor called Hairy is one of the first activated during segment development in the fruitfly Drosophila melanogaster. Misregulation of Hairy and related factors is associated with cancer and developmental defects across species. Mutations in the gene that codes Hairy lead to overexpression of genes involved in development, but it is not clear whether Hairy normally represses those genes directly, or through intermediaries. To find the answer, Susan Parkhurst and colleagues at the Fred Hutchinson Cancer Research Center in Seattle, Washington, set out to identify Hairy's direct targets.

The researchers found a total of 59 genes bound directly by the Hairy protein in cultured Drosophila cells called Kc cells and in embryos collected at the peak of Hairy expression during Drosophila segmentation. Because they searched approximately half of the expected Drosophila genome, the researchers estimate that they identified roughly half of all Hairy target genes. The list included genes known to act during segmentation, as expected, as well as many others with roles in cell division, growth, and shape.

Of the 59 Hairy targets identified, only one appeared in both Kc cells and embryos. The lack of overlap may reflect a difference in developmental stage between the Kc cells, which are thought to be precursors to neurons, and the relatively undifferentiated embryos, and suggests that Hairy's role changes with context, such as the stage of development or tissue type. But Hairy doesn't act alone. Like most transcription factors, Hairy requires assistant proteins, called cofactors, to do its job. The availability of Hairy's known cofactors—Groucho, Drosophila C-terminal binding protein (dCtBP), and Drosophila Sir2 (dSir2)—may help to set the tempo and timing of Hairy's repression of genes.

Groucho was thought to be Hairy's main assistant, but Parkhurst and colleagues found that only one of the identified Hairy target genes bound Groucho in Kc cells. The majority of Hairy targets overlapped with those of dCtBP, most often in combination with dSir2. All of the cofactors also bound to non-Hairy targets, suggesting that they assist other transcription factors as well.

Next, Parkhurst's lab plans to explore the biological functions of the 59 Hairy target genes, to see how they help Hairy coordinate development. Meanwhile, the list of known Drosophila genes has grown 2-fold to include almost all of the expected genome. Repetition of these experiments with the expanded gene set could identify most or all of Hairy's target genes.

The current results suggest that Hairy plays roles in segmentation, cell division, and tissue formation that evolve as an organism develops. Differences in cofactor involvement could help regulate Hairy's repression of genes. This paper demonstrates a powerful technique to explore how developing embryos keep gene expression in step.

